# Acellular dermal matrix alone or in combination with subacromial bursa and humeral bone marrow concentrate for augmentation of massive rotator cuff tear repair: a double-blind randomized controlled trial protocol (MODA)

**DOI:** 10.3389/fmed.2026.1724038

**Published:** 2026-03-06

**Authors:** Marco Cavallo, Luca Solaro, Fabio Tortorella, Valentina Brunello, Davide Melandri, Stefania Pagani, Lucia Martini, Enrico Guerra, Matilde Tschon

**Affiliations:** 1 Shoulder and Elbow Surgery, IRCCS Istituto Ortopedico Rizzoli, Bologna, Italy; 2 Burn Center Operative Unit and Emilia Romagna Regional Skin Bank, Department of Surgery and Trauma Center, Bufalini Hospital, AUSL della Romagna, Cesena, Italy; 3 Surgical Sciences and Technologies, IRCCS Istituto Ortopedico Rizzoli, Bologna, Italy

**Keywords:** acellular dermal matrix, arthroscopic augmentation, humeral bone marrow concentrated, massive rotator cuff tear repair, orthobiologics, subacromial bursa

## Abstract

**Background:**

Massive rotator cuff tears are challenging injuries, and there is currently a clinical need to improve treatment outcomes and reduce the re-tear rate. In recent years, both mechanical augmentation with acellular matrices, such as acellular dermal matrix and orthobiologics, including concentrated humeral bone marrow or subacromial bursal tissue, have been proposed to enhance clinical outcomes of the rotator cuff repair.

**Methods:**

Seventy-two patients (aged 18–65 years) with repairable large to massive rotator cuff tears involving supraspinatus and infraspinatus tendons are randomized in a 1:1 ratio to receive either acellular dermal matrix alone (Group A: mechanical augmentation) or a combination of acellular dermal matrix enriched with bone marrow concentrated from the humeral head and subacromial bursal tissue (Group B: mechanical and biological augmentation). The primary outcome is the re-tear rate at 1 year follow-up, assessed via MRI using Sugaya classification. Secondary outcomes include clinical and functional evaluations using the Constant-Murley, Visual Analogue Scale, PASS and DASH scores at 1, 3, 6, and 12 months. Work loss and restricted activity days and adverse events are also recorded. Biological and biomechanical characterization of acellular dermal matrix, concentrated humeral bone marrow and subacromial bursa are measured and correlated with clinical outcomes to find parameters affecting the tendon repair.

**Expected outcomes:**

A more robust tendon-to-bone reattachment, vascularization and cell colonization are expected to face to patient’s functional demand and to diminish the psychological and economic burden resulting from re-tears, revisions, additional rehabilitation and missed work. The use of decellularized human matrix provides benefits, since it derives from donation of human tissue, with low production and distribution costs in comparison with commercially available acellular matrices.

**Clinical trial registration:**

[clinicaltrials.gov], identifier [NCT05855759].

## 1 Background

Rotator Cuff (RC) tears are among the most frequent soft-tissue injuries affecting the shoulder, and can lead to pain, disability and premature osteoarthritis. RC repair is characterized by a highly variable re-tear rate, ranging from 24% up to 94% depending on age, chronicity, tear size, and repair technique, among other factors (1). The treatment of massive RC tears is particularly challenging because of poor tendon quality, hypo-vascularization and the formation of a repair scar tissue that is not biomechanically competent (1, 2). During recent years, the clinical interest in biological acellular matrices is increasing because of their capacity in providing the ideal biologic and structural milieu for tissue integration and regeneration. Matrices of animal and human origins have been investigated in preclinical researches and low evidence clinical studies with variable results and the lack of conclusive proof of efficacy (1). To date, none of the available augmentation techniques have achieved the regeneration of a tendon-bone attachment that is structurally and functionally non-inferior to the native enthesis. An improvement in surgical repair and cost-effectiveness is reported in a randomized controlled trial with a collagen based bioinductive implant compared to repair alone (3). Augmentation with cross-linked acellular porcine dermal patch is reported to provide a non-inferior failure load and reduced inflammatory response compared to porcine small intestine submucosa patch in an ovine model study at 2 years follow-up (4).

Instead, augmentation of large-to-massive RC tears with human dermal allografts is associated with superior functional and structural outcome when compared with conventional primary repair (5).

In this scenario, an acellular dermal matrix (ADM) from human donors is developed (PCT n. IB/2008/002753): ADM is obtained from multi-organ and/or multi-tissue donors, the decellularization process removes the cellular components responsible for rejection in the recipient, while preserving its structural, biomechanical, and biological integrity, which confer bioactivity, biomechanical competence, and regenerative capacity. The patented ADM scaffold is used for many applications in medicine and surgery (tendon, ankle reconstruction, breast and gynecologic surgery, dermatology, dentistry, urology and ophthalmology), with good results concerning safety and clinical outcomes (6–10). ADM is extensively characterized and tested in various steps of preclinical *in vitro* and *in vivo* experimentations (11–13) and in a previous work transplanted into seven patients affected by massive **R**C lesions: at 1 year of follow up, all patients showed an improvement in symptoms and no side effects, inflammatory or septic complications occurred (2). From these preliminary results, the rationale for this trial is to gain evidence-based and robust data regarding ADM implantation in a larger cohort of patients, thanks to excellent mechanical and biological properties. Moreover, a growing interest is focusing on ADM, aiming to combine the biological potential to be integrated into the patient’s repaired tendon and mechanical strength, to preserve the repaired tendon from early failure.

The use of orthobiologics is gaining interest due to the biological eutrophic effects and the homeostatic influence of several products, ranging from blood-derivatives as platelet-rich-plasma to minimally manipulated mesenchymal stromal cells (MSCs) or connective tissue progenitors (14).

Hernigou et al. (15) reports structural healing improvements evaluated with ultrasound and MRI at 6 months and significant lower re-tears at 10 years when RC repairs are augmented with bone marrow aspirate concentrate. More recently, a Randomized Controlled Trial is performed to compare outcomes after arthroscopic RC repair with and without bone marrow aspirate augmentation. At one year, the control group shows significantly greater evidence of rotator cuff re-tear on MRI according to Sugaya classification (57% vs. 18%; *P* < 0.001), but failure rates and functional outcomes are comparable at final follow-up (16).

Proximal humerus is reported as a suitable source of MSCs, with samples of bone marrow aspirate from the proximal humerus yielding a significantly higher amount of Colony Forming Units (CFUs) when compared with samples of bone marrow obtained from the ilium (17, 18). From our preliminary studies about 10−250 × 10^6^ adherent mesenchymal stromal cells/ml of humeral bone marrow have been isolated, that are ranges in line with literature and the manufacturer’s product specifications (19).^1^

Subacromial bursa (SAB) is also identified as a source of connective tissue progenitors and vasculature supporting factors, and SAB-derived cells showed mesenchymal potential and higher proliferation compared with cells derived from concentrated bone marrow (20, 21). Moreover, the use of SAB is reported for biological augmentation of RC healing with good results, both as an intact tissue, or as a processed tissue in combination with other injective products (22–24). From culturing the subacromial bursal tissues we obtained about 20–80 CFU/well by seeding 2 × 10^3^ cells even in line with scientific literature available (20, 21).

A clinical trial on ADM arthroscopic augmentation for RC massive tear repair has never been performed as well as, to the best of our knowledge, on the combination of acellular human matrices with orthobiologic products derived from the patient’s shoulder anatomic district, which could improve outcomes reducing the re-tear rate.

## 2 Hypothesis and aims

The hypothesis of our study is that the combination of mechanical and biological augmentation could potentially lead to a superior quality of the repair, with improved functional and structural results and lower re-tears and failure rate. The secondary hypothesis is that there will be relationships among the specific biological characteristics of ADM, SAB and concentrated humeral bone marrow (cHBM) from patients and the post-operative clinical outcomes.

The main aim is to evaluate the therapeutic efficacy in terms of re-tear rate at 12 months from arthroscopic implantation of ADM in association with orthobiological stimuli for the augmentation of massive RC repair in comparison with the ADM alone. The secondary aim is the *in vitro* biological characterization of SAB and cHBM, as surgical discharge materials from each patient, and biomechanical competence of ADM to determine specific correlations with the obtained clinical outcome investigations.

## 3 Methods

### 3.1 Study setting

A double-blinded randomized controlled trial (RCT) is designed to compare the efficacy of ADM in association with orthobiological stimuli, the one-step concentrated humeral bone marrow and vascularized subacromial bursa versus ADM alone for the augmentation of massive RC tears arthroscopic repair. This study is a single-center, double-blind RCT conducted in a highly specialized center for orthopedic surgery, the IRCCS Rizzoli Orthopedic Institute (Bologna, Italy), and performed by the close collaboration between the Unit dedicated exclusively to Shoulder and Elbow Surgery and the Unit of Surgical Sciences and Technologies devoted to preclinical biological and biomechanical investigations. The Regional Skin Bank (AUSL Romagna, Cesena, Italy) produces ADM in Good Manufacturing Practice certified environments for transplant use. The trial is approved by the Ethical Committee of Area Vasta Emilia Centro (CE AVEC) (protocol number 0004860) and registered at clinicaltrials.gov (NCT05855759).

After signing a study specific informed consent, each participant is assigned randomly with ratio 1: 1 to RC repair with ADM implantation (Group A), or ADM implantation with orthobiologics, consisting of autologous cHBM and SAB (Group B).

### 3.2 Eligibility criteria

Patients are recruited according to the following criteria.

Inclusion criteria:

- Male or female patients, aged between 18 and 65 years.
- Large to Massive Rotator Cuff tear involving supraspinatus and infraspinatus tendons with tendon retraction ≤ Thomazeau 3 and fatty infiltration ≤ Goutallier 3 at pre-op MRI.
- Possibility to obtain tendons reduction at footprint and complete repair.
- Patients’ ability and consent to participate in clinical and radiological follow-up.
- Signature of informed consent.

Exclusion criteria:

- Shoulder Osteoarthitis, even mild according to Samilson.
- Adhesive capsulitis.
- Symptomatic acromioclavicular arthritis.
- Previous RC repair in the affected shoulder.
- Current or past hematological disorders.
- Chronic steroid assumption or comorbidities affecting healing.
- Patients with malignancy, rheumatic diseases. uncompensated endocrine diseases.
- Patients abusing alcoholic beverages or drugs.
- Pregnancy and lactation.
- Inability to cope with post-op rehabilitation regimen.

### 3.3 Allocation and randomization

A total of 72 eligible patients are allocated to receive either a combination of mechanical (ADM) and biological (cHBM + SAB) augmentation after RC repair or mechanical augmentation with ADM alone, in a 1:1 ratio (36 patients for each group of treatment) based on a computer-generated randomization.

The randomization list is created using an online randomization list generator^2^ with a permuted block of variable length from 2 to 6. Enrolled patients are randomly assigned to the massive RC repair augmented with autologous cHBM, SAB and ADM arm (group B) or to the ADM augmentation alone (group A), as comparator arm, following a pre-determined randomization list. Patients and outcome assessors are blinded, while the surgeon knows the group assignation of the patient the day of surgery, opening a dedicated closed envelope.

### 3.4 ADM procurement

Acellular dermal matrix harvesting, processing, and distribution are performed according to national rules on tissues for transplantation. Briefly, samples of human dermis are harvested from multi-organ and multi-tissue donors, processed for decellularization (PCT n. IB/2008/002753) and stored in liquid nitrogen at −180 °C. The day before surgery, ADM is defrosted and conserved at a temperature of 4 °C until its use.

### 3.5 Intervention

All patients are treated by a single orthopedic surgeon with established experience in sports medicine shoulder surgery and orthobiologic procedures. The procedure is performed in a single step in the operating room with patients in beach-chair position under general anesthesia. The affected shoulder is sterilely prepared and draped as for standard arthroscopic RC repair.

In all patients a standard shoulder arthroscopy is performed, and all patients undergo a comprehensive arthroscopic evaluation to confirm the RC tear characteristics and assess repairability.

#### 3.5.1 Group A

After a standard diagnostic arthroscopy, RC repair is carried out. Posterosuperior RC repair is performed using non-metallic anchors and, when necessary, subscapularis repair is also performed. If biceps pathology is identified during diagnostic arthroscopy, arthroscopic biceps tenotomy or tenodesis is concurrently performed. Once the arthroscopic repair is completed, the ADM is inserted into the subacromial space via an accessory lateral portal and sutured with non-metallic anchors and suture tape and on top of the repaired RC. Standard closure of the portals is then performed and an abduction sling is positioned.

#### 3.5.2 Group B

For Group B patients, before starting the arthroscopic procedure, a stab incision is performed, a diamond tip trocar is inserted into the proximal humeral epiphysis and humeral bone marrow is harvested using two 30 ml syringes coated with heparin for a total of 60 ml. The bone marrow harvesting time requires approximately 10 min, and while on the surgical field the rotator cuff repair begins, the bone marrow isconcentrated by an automatic close system (Harvest BMAC, Terumo BCT Inc., with a dedicated harvesting and concentrating disposable kit costing approximately 1500 Euros). After the 15-min working cycle a total of 10 ml of bone marrow autologous concentrated (cHBM) is available.

In Group B, at the beginning of the shoulder arthroscopy, approximately 4 mg of SAB are harvested, processed with chopping technique and stored in sterile saline solution for subsequent implant. The cuff is then repaired and the graft positioned, as previously described for group A. After the completion of the mechanical augmentation and suture of the arthroscopic portals, in group B patients, 8 ml cHBM and about 3 ml of the processed SAB are injected into the repair site. For each group B patient, 2 ml cHBM and 1 ml SAB samples, as residual materials from the operating room, are sent to the laboratory for the count of mesenchymal stromal cells, clonogenic ability by colony forming unit-fibroblast test, phenotypical characterization by flow-cytometry and trilineage differentiation. From each group, ADM samples are tested for mechanical and biological competences: maximum load, tensile strength, Young’s modulus, stiffness, growth factors release and extracellular matrix protein contents are measured.

### 3.6 Post-operative protocol

Patients are normally discharged on postoperative day 1, based on patient condition and pain control is prescribed as needed. All patients undergo the same rehabilitative protocol, consisting in 1-month of sling abduction with no shoulder movements. The second month post-operative patients gradually dismiss the sling, and start passive movements with physiotherapist for 2 weeks, followed by active exercises avoiding reinforce for the next 6 weeks. Exercises for muscular strengthening are allowed 3 months after surgery, along with full recovery of daily and working activities.

### 3.7 Outcomes

#### 3.7.1 Primary outcome

The primary outcome is the re-tear rate on MRI at 1 year follow up, with the rational of verifying the integrity of the repaired tendon after several months of daily and working regular life, and detecting scaffold integration at the implant site. The integrity of the repair in post-operative MRI will be assessed with Sugaya classification.

#### 3.7.2 Secondary clinical outcomes

Secondary outcomes include clinical and functional assessments, performed pre-operatively and at 1-, 3-, 6- and 12-months post-op using the Constant-Murley, Visual Analogue Scale (VAS), PASS and DASH scores. Secondary outcomes also include work loss and restricted activity days and adverse events.

#### 3.7.3 Secondary research outcomes

The *in vitro* biological characterization of orthobiologics from each patient, cHBM and SAB, are evaluated in term of cellular yield, proliferation ability, mesenchymal potential, surface marker expression and multilineage differentiation; SAB is also characterized by histological and gene expression approaches to define its characteristics for angiogenetic, inflammatory and regenerative factors. Finally, ADM is evaluated for its contribution in terms of growth factors release, extracellular matrix contents, and biomechanical properties (maximum load, tensile strength, Young’s modulus and stiffness). The biological characterizations of cHBM and SAB and the biomechanical characterization of ADM are used in correlation with the clinical ourcomes, in order to identify any parameter that might have influenced the clinics.

### 3.8 Participant timeline

A screening of potential candidates is performed by the members of the Shoulder and Elbow Unit among patients affected by a massive RC tear. If eligibility is possible, patients are informed of the study protocol and they are offered to enter in the study, with adequate time to decide. If they accept to enter the clinical trial, they sign informed consent and complete the baseline clinical scores. Follow-up assessments are performed at 1-, 3-, 6-, and 12- months post-operatively with clinical scores and at 12- months post-operatively with MRI.

Participant timeline is outlined in Table 1.

**TABLE 1:** Participant timeline.

Procedure	Pre-op	1-month follow-up	3-month follow-up	6-month follow-up	12-month follow-up
Eligibility and informed consent	X	–	–	–	–
CMS	X	X	X	X	X
VAS	X	X	X	X	X
DASH	X	X	X	X	X
PASS	X	X	X	X	X
MRI	X	–	–	–	X
AE reporting	–	X	X	X	X

CMS, Constant-Murley Score for clinical and functional shoulder evaluation; VAS, Visual Analogue Scale for pain intensity; DASH, Disabilities of the Arm, Shoulder, and Hand for disability and symptoms of the upper limb; PASS, Patient Acceptable Symptom State evaluates whether patients perceive their state as satisfactory; MRI, Magnetic Resonance Imaging with Sugaya classification for re-tear assessment; AE, adverse events (traumas, falls…) recorded at each Follow up.

### 3.9 Adverse events and assessment process

Adverse events are monitored throughout the study and at clinical follow-up evaluations. Standard safety and efficacy monitoring is performed through visits. Patients are requested to report any adverse events to the research staff. Every adverse event is recorded in the patient case report form (CRF). Serious adverse events are considered those resulting in death or being life-threatening, requiring hospitalization or intervention to prevent permanent impairment or damage; they are reported in accordance with the requirements of the Ethical Committee.

### 3.10 Data collection methods

Patients’ clinical records (informed consent, hospital admittance, interventions, radiologic imaging and follow-up) are registered in SIR2020 (v. 3.1.0, Engineering Ingegneria Informativa S.p.A, Italy). The data manager, after having allocated patients following the randomization list, attributes a numerical code to patients, so that sensitive data will be made pseudo-anonymized and used in compliance with the in-force legislation regarding privacy. Study data of patient’s records are collected and managed using REDCap electronic data capture tools hosted at Yale University and in the MODA database with the assigned numerical code. Data are accessible only to the PI, co-PI and their authorized collaborators or to monitoring or auditing procedures or to the Ethics Committee and relevant health authorities. Results from *in vitro* activities obtained from each experiment are saved in an e-CRF form in the MODA folder with appropriate password and limited access to authorized personnel.

### 3.11 Data management

Study data are stored in a password-protected MODA folder on a server that is hosted at the Rizzoli Orthopedic Institute. Data transfer is encrypted with all data de-identified. Only authorized trained research personnel specifically dedicated to data handling can access the database and ensure the correspondence of the electronic data with the original paper-based clinical scores and medical charts.

### 3.12 Statistical analysis

To identify the minimum patient sample size, an *a priori* power analysis is performed (G*Power 3.1.9.6v software, University of Kiel, D) based on a previous study (2), which used the same ADM on *n* = 5 patients, obtaining a re-tear rate of 40%. Furthermore, Cai et al. (24) obtained a re-tear rate of 13.7% by combining the suture with orthobiological augmentation. The hypothesis of the present study was that the addition of orthobiologicals would result in a re-tear rate of 10%, thus yielding an OR value of 6.

By considering an odds ratio (OR) = 6.0 for re-tear rate between ADM-orthobiologics and ADM alone, 95% confidence and 0.80 power (Fisher’s exact test, onetail) *n* = 36 for both groups is computed. This patient size considers 20% of drop-out patients.

All demographics, clinical, MRI, biological, biomechanical, histological, follow up clinical data are recorded in the electronic dataset for further statistical analysis. The statistical analysis of the results is conducted using SPSS/PC + Statistics TM (SPSS Inc., United States) or the R software (R Core Team, 2021^3^) and the related packages necessary for conducting the analysis such as ggplot2 (25) for graphical data representation. A *p*-value < 0.05 is considered statistically significant.

### 3.13 Data monitoring

A central project data manager is tasked to perform data quality control on all collected data. An *ad interim* report and a final report are foreseen, to be submitted to the Ministry of Health who funded the project. A further project auditing is performed by another independent entity of the Institution, the Clinical Trial Center. The final study report is also sent to the Ethic Committee.

## 4 Discussion

This is the first randomized, double-blind, and controlled trial comparing the efficacy of human acellular dermal matrix (ADM) alone or combined with humeral bone marrow aspirate concentrate (cHBM) and subacromial bursa (SAB) for re-tear prevention in treatment of RC Repair. Patients are analyzed using Patient Reported Outcome Measures (PROMs), objective measures and MRI examination, and biomarker evaluation. Orthobiologics and ADM are analyzed for all baseline characteristics, in terms of number of cells, regenerative potentials and biomechanical competence, and correlated with clinical outcomes.

This study can clarify the benefits and limitations of the newly proposed dermal allograft for RC tears and the role of associated orthobiologics augmentation, providing precious indications for the clinical practice.

## Footnotes

1
https://www.terumopenpol.com/wp-content/uploads/2019/12/BONE-MARROW.pdf

2
www.sealedenvelope.com

3
https://www.R-project.org

## References

[1] Kovacevic D Suriani RJ Levine WN Thomopoulos S. Augmentation of rotator cuff healing with orthobiologics. *J Am Acad Orthop Surg.* (2022) 30:e508–16. 10.5435/JAAOS-D-20-01011 34932515 PMC8881347

[2] Rotini R Marinelli A Guerra E Bettelli G Castagna A Fini M Human dermal matrix scaffold augmentation for large and massive rotator cuff repairs: preliminary clinical and MRI results at 1-year follow-up. *Musculoskelet Surg.* (2011) 95:S13–23. 10.1007/s12306-011-0141-8 21691735

[3] Rognoni C Nherera LM Garofalo R Guerra E Longo UG Taverna E Economic evaluation of a bioinductive implant for the repair of rotator cuff tears compared with standard surgery in Italy. *Adv Ther.* (2023) 40:5271–84. 10.1007/s12325-023-02686-9 37759150 PMC10611596

[4] Nicholson GP Breur GJ Van Sickle D Yao JQ Kim J Blanchard CR. Evaluation of a cross-linked acellular porcine dermal patch for rotator cuff repair augmentation in an ovine model. *J Shoulder Elbow Surg.* (2007) 16:S184–90. 10.1016/j.jse.2007.03.010 17574876

[5] Ferguson DP Lewington MR Smith TD Wong IH. Graft utilization in the augmentation of large-to-massive rotator cuff repairs: a systematic review. *Am J Sports Med.* (2016) 44:2984–92. 10.1177/0363546515624463 26847487

[6] Folli S Curcio A Melandri D Bondioli E Rocco N Catanuto G A new human-derived acellular dermal matrix for breast reconstruction available for the European market: preliminary results. *Aesthetic Plast Surg.* (2018) 42:434–41. 10.1007/s00266-017-1069-7 29302735

[7] Melandri D Marongiu F Carboni A Rubino C Razzano S Purpura V A new human-derived acellular dermal matrix for 1-stage coverage of exposed tendons in the foot. *Int J Low Extrem Wounds.* (2020) 19:78–85. 10.1177/1534734619884422 31679415

[8] Lughi M Campagna A Purpura V Bondioli EA. New treatment for the reconstruction of the medial compartment of the ankle: the combined use of biological materials. *Joints.* (2021) 7:228–32. 10.1055/s-0041-1730380 34235391 PMC8253609

[9] Bondioli E Purpura V Orlandi C Carboni A Minghetti P Cenacchi G The use of an acellular matrix derived from human dermis for the treatment of full-thickness skin wounds. *Cell Tissue Bank.* (2019) 20:183–92. 10.1007/s10561-019-09755-w 30767153

[10] Ghetti M Papa V Deluca G Purpura V Ruscelli P Melandri D Histological and ultrastructural evaluation of human decellularized matrix as a hernia repair device. *Ultrastruct Pathol.* (2018) 42:32–8. 10.1080/01913123.2017.1365788 29192810

[11] Giavaresi G Bondioli E Melandri D Giardino R Tschon M Torricelli P Response of human chondrocytes and mesenchymal stromal cells to a decellularized human dermis. *BMC Musculoskelet Disord.* (2013) 14:12. 10.1186/1471-2474-14-12 23294867 PMC3547812

[12] Bondioli E Fini M Veronesi F Giavaresi G Tschon M Cenacchi G Development and evaluation of a decellularized membrane from human dermis. *J Tissue Eng Regen Med.* (2014) 8:325–36. 10.1002/term.1530 22689414

[13] Fini M Bondioli E Castagna A Torricelli P Giavaresi G Rotini R Decellularized human dermis to treat massive rotator cuff tears: in vitro evaluations. *Connect Tissue Res.* (2012) 53:298–306. 10.3109/03008207.2011.649929 22172074

[14] Eliasberg CD Trinh PMP Rodeo SA. Translational research on orthobiologics in the treatment of rotator cuff disease: from the laboratory to the operating room. *Sports Med Arthrosc Rev.* (2024) 32:33–7. 10.1097/JSA.0000000000000395 38695501

[15] Hernigou P Flouzat Lachaniette CH Delambre J Zilber S Duffiet P Chevallier N Biologic augmentation of rotator cuff repair with mesenchymal stem cells during arthroscopy improves healing and prevents further tears: a case-controlled study. *Int Orthop.* (2014) 38:1811–8. 10.1007/s00264-014-2391-1 24913770

[16] Cole BJ Kaiser JT Wagner KR Sivasundaram L Otte RS Tauro TM Prospective randomized trial of biologic augmentation with bone marrow aspirate concentrate in patients undergoing arthroscopic rotator cuff repair. *Am J Sports Med.* (2023) 51:1234–42. 10.1177/03635465231154601 36811557

[17] Otto A Muench LN Kia C Baldino JB Mehl J Dyrna F Proximal humerus and ilium are reliable sources of bone marrow aspirates for biologic augmentation during arthroscopic surgery. *Arthroscopy.* (2020) 36:2403–11. 10.1016/j.arthro.2020.06.009 32554079

[18] LaPrade RF Murray IR. Editorial commentary: bone marrow aspirate concentrate: time to harvest locally? *Arthroscopy.* (2020) 36:2412–4. 10.1016/j.arthro.2020.07.015 32891243

[19] Morikawa D Johnson JD Kia C McCarthy MBR Macken C Bellas N Examining the potency of subacromial bursal cells as a potential augmentation for rotator cuff healing: an in vitro study. *Arthroscopy.* (2019) 35:2978–88. 10.1016/j.arthro.2019.05.024 31629585

[20] Morikawa D Hawthorne BC McCarthy MBR Bellas N Johnson JD Trudeau MT Analysis of patient factors affecting in vitro characteristics of subacromial bursal connective tissue progenitor cells during rotator cuff repair. *J Clin Med.* (2021) 10:4006. 10.3390/jcm10174006 34501453 PMC8432549

[21] Morikawa D LeVasseur MR Luczak SB Mancini MR Bellas N McCarthy MBR Decreased colony-forming ability of subacromial bursa-derived cells during revision arthroscopic rotator cuff repair. *Arthrosc Sports Med Rehabil.* (2021) 3:e1047–54. 10.1016/j.asmr.2021.03.010 34430884 PMC8365201

[22] Bhatia DN. Arthroscopic bursa-augmented rotator cuff repair: a vasculature-preserving technique for subacromial bursal harvest and tendon augmentation. *Arthrosc Tech.* (2021) 10:e1203–9. 10.1016/j.eats.2021.01.013 34141532 PMC8185525

[23] Muench LN Uyeki CL Mancini MR Berthold DP McCarthy MB Mazzocca AD. Arthroscopic rotator cuff repair augmented with autologous subacromial bursa tissue, concentrated bone marrow aspirate, platelet-rich plasma, platelet-poor plasma, and bovine thrombin. *Arthrosc Tech.* (2021) 10:e2053–9. 10.1016/j.eats.2021.05.008 34504743 PMC8417132

[24] Cai YZ Zhang C Jin RL Shen T Gu PC Lin XJ Arthroscopic rotator cuff repair with graft augmentation of 3-dimensional biological collagen for moderate to large tears: a randomized controlled study. *Am J Sports Med.* (2018) 46:1424–31. 10.1177/0363546518756978 29533674

[25] Wickham H. *ggplot2: Elegant Graphics for Data Analysis.* New York, NY: Springer New York (2009).

